# PKC regulation of ion channels: The involvement of PIP_2_

**DOI:** 10.1016/j.jbc.2022.102035

**Published:** 2022-05-16

**Authors:** Kirin D. Gada, Diomedes E. Logothetis

**Affiliations:** 1 Department of Pharmaceutical Sciences, Bouvé College of Health Sciences and College of Science, Northeastern University, Boston, Massachusetts, USA; 2 Department of Chemistry and Chemical Biology, Bouvé College of Health Sciences and College of Science, Northeastern University, Boston, Massachusetts, USA; 3 Center for Drug Discovery, Bouvé College of Health Sciences and College of Science, Northeastern University, Boston, Massachusetts, USA

**Keywords:** PKC, phosphatidylinositol (4,5) bisphosphate, ion channel, phosphorylation, voltage-gated potassium channel, inwardly rectifying potassium channels, transient receptor potential channels, ASIC, acid-sensing ion channel, BIM, bisindolylmaleimide, CaCC, Ca^2+^-activated chloride channel, Ca_V_, voltage-gated calcium, ClC, chloride channel, CFTR, cystic fibrosis transmembrane conductance regulator, cPKC, conventional PKC, DAG, diacylglycerol, diC8-PIP_2_, dioctanoyl PI(4,5)P_2_, DRG, dorsal root ganglia, ENaC, epithelial Na^+^ channel, GIRK, G-protein-gated inwardly rectifying K^+^ channel, K_2P_, two-pore domain potassium, K_Ca_, calcium-activated potassium, K_ir_, inwardly rectifying potassium, K_V_, voltage-gated potassium, LQTS, long QT syndrome, MD, molecular dynamics, MOR, μ-opioid receptor, MS, mass spectrometry, nPKC, novel PKC, PdBu, phorbol 12,13-dibutyrate, PI(4,5)P_2_ or PIP_2_, phosphatidylinositol (4,5) bisphosphate, PMA, phorbol 12-myristate 13-acetate, SUR, sulfonylurea receptor subunit, TMTX, thymeleatoxin, TRP, transient receptor potential, TRPC, TRP canonical, TRPM, TRP melastatin, TRPP, TRP polycystic, TRPV, TRP vanilloid, TTX, tetrodotoxin, VSD, voltage sensor domain

## Abstract

Ion channels are integral membrane proteins whose gating has been increasingly shown to depend on the presence of the low-abundance membrane phospholipid, phosphatidylinositol (4,5) bisphosphate. The expression and function of ion channels is tightly regulated *via* protein phosphorylation by specific kinases, including various PKC isoforms. Several channels have further been shown to be regulated by PKC through altered surface expression, probability of channel opening, shifts in voltage dependence of their activation, or changes in inactivation or desensitization. In this review, we survey the impact of phosphorylation of various ion channels by PKC isoforms and examine the dependence of phosphorylated ion channels on phosphatidylinositol (4,5) bisphosphate as a mechanistic endpoint to control channel gating.

The ability of kinases to modulate ion channels is a prevalent theme in the literature. Phosphorylation is believed to have the ability to alter channel open probability, gating, voltage dependence, desensitization, and in some cases, permeability (1). Nature’s use of kinases as “molecular switches” is a versatile mechanism used in numerous contexts throughout biology.

The human kinome consists of 518 kinases that serve multiple purposes and are an intricate part of every life process. Kinases are divided into eight broad groups. These groups are tyrosine kinase, tyrosine kinase-like, STE (STE20, STE11, and STE7 related), casein kinase 1, AGC (PKA-related, protein kinase G–related, and PKC-related), Ca^2+^/calmodulin-dependent kinases, CMGC (Cdk-, MAPK-, GSK-related, and Cdk–like-related), and receptor guanylyl cyclase (2).

Phosphorylation of ion channels by PKC has been studied for decades. PKC is a family of serine/threonine kinases that is involved in signal transduction through the second messenger diacylglycerol (DAG). The PKC family is divided into conventional PKC (cPKC), atypical PKC, and novel PKC (nPKC) enzymes based on the binding preferences of their regulatory domains (3). All PKCs share the same basic structure with the membrane-targeting segments residing in the NH_2_-terminal domain and the ATP- and substrate-binding regions in the COOH-terminal domain (4) (Fig. 1*A*). The catalytic domain is contained in the C4 region of the enzyme while C3 possesses the ATP-binding site (4). The regulatory subunit of cPKCs (α, β_I_, β_II_, and γ isoforms) contains a C1 region, which is cysteine rich and binds to DAG. A Ca^2+^ sensing, C2 region is also sensitive to acidic lipids. cPKCs can be activated by phosphatidylserine, DAG, and an increase in Ca^2+^. cPKCs are also called Ca^2+^-sensitive PKCs and are activated after calcium binds to the C2 region and increases their affinity for phosphatidylserine at the cell surface, which in turn allows the binding of DAG leading to full activation of the enzymes (5). Canonical activation of cPKCs through the hydrolysis of phosphatidylinositol (4,5) bisphosphate (PI(4,5)P_2_ or PIP_2_) is shown in Figure 1*B*.

**Figure 1:**
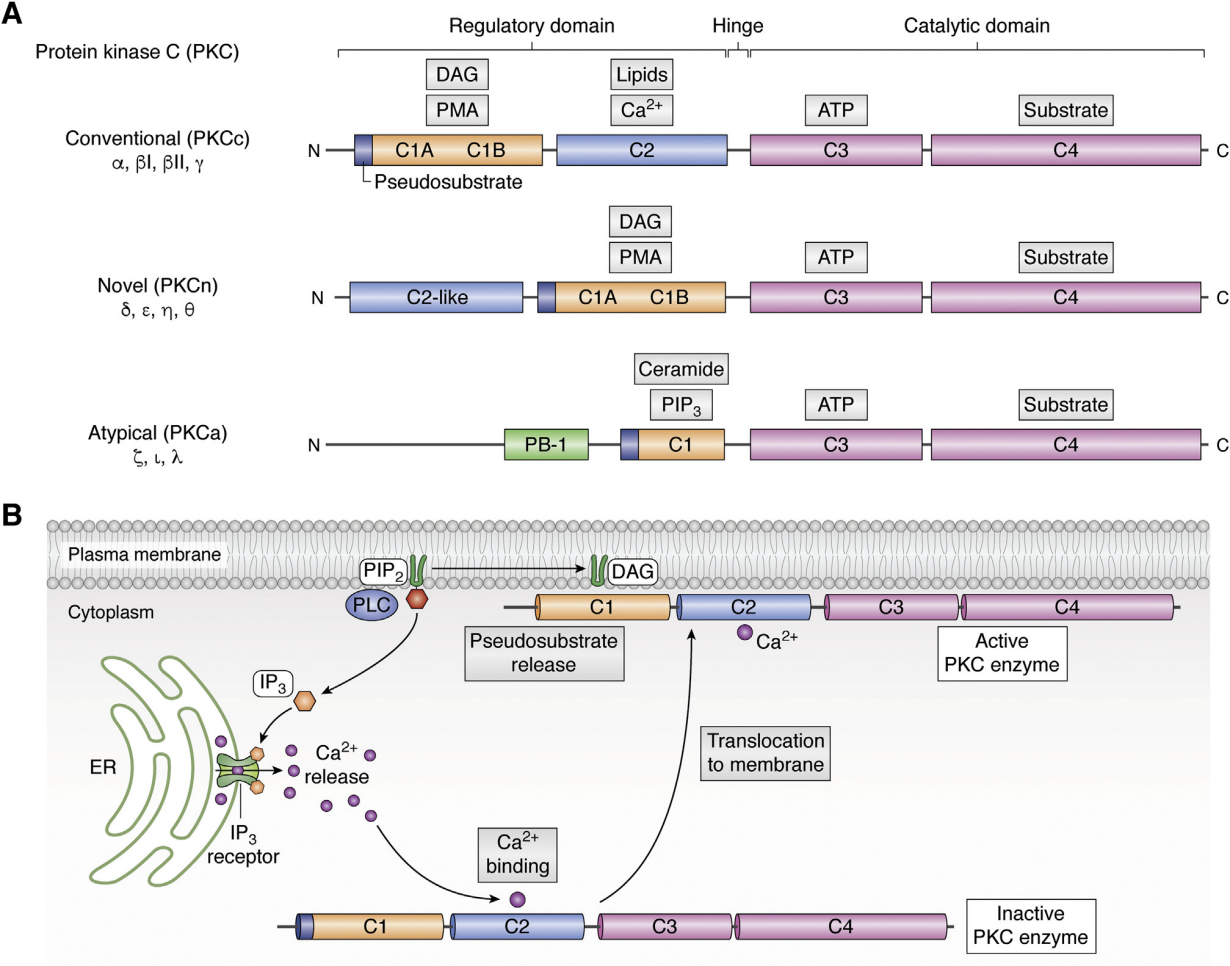
**Schematic representation of PKC isozyme structures and conventional PKC (cPKC) activation**. *A*, C1 (*orange*) domains in the N-terminal region bind diacylglycerol (DAG) and phorbol esters like phorbol myristate acetate (PMA). The C2 domain (*blue*) binds anionic lipids and calcium. The pseudosubstrate region (*blue*), which is located on the N-terminal end of the C1 domain, is a sequence of amino acids that mimics a substrate; the C1 domain in aPKCs binds PIP_3_ or ceramide. The C-terminal catalytic domain contains the ATP-binding domain C3 (*magenta*) and the substrate binding domain C4 (*magenta*). *B*, hydrolysis of PIP_2_ by phospholipase C releases two second messengers, DAG and inositol triphosphate (IP_3_). IP_3_ releases calcium from the endoplasmic reticulum (ER), which binds to the C2 domain of cPKCs while DAG binds to the C1 domain; the pseudosubstrate is released resulting in activation of cPKC and translocation to the cell membrane. (*Purple circles*, Ca^2+^; *green*, IP_3_ receptor in ER). aPKC, atypical PKC; PIP_2_, phosphatidylinositol (4,5) bisphosphate.

The nPKCs, δ, ε, η, and θ, are structurally similar to cPKCs, but they do not need Ca^2+^ for activation and, unlike cPKCs, lack the Ca^2+^-sensing ability through the C2 domain (Fig. 1). However, this subtype has a higher affinity for DAG and is activated by the binding of DAG to the C1 domain (5). The levels of DAG in the cell are tightly regulated. PKCs sense the small increases in DAG following receptor-mediated signal transduction.

The structure of atypical PKCs—ζ, ι, and λ—is significantly different from cPKCs and nPKCs (4), as they are both insensitive to DAG and lack the Ca^2+^-sensing C2 region (Fig. 1). These PKCs are thought to be sensitive to activation by lipid-derived second messengers like arachidonic acid (4).

PKC enzymes differ in their tissue distribution and specificity. The species and tissue-specific expression of PKC enzymes is beyond the scope of this review but has been reviewed by others (6). Most cPKCs and nPKCs, especially PKCα, βI, βII, and PKCε, respectively, are found to be expressed in cardiac tissue (4). PKCs are involved in the phosphorylation of kinases, growth factors, ion channels, and the gene expression machinery in cardiac tissue (4).

PKC isozymes are heavily involved in tumorigenesis. The bioelectrical properties of cancer cells are distinct from normal cells. The depolarized membrane potential of cancer cells is thought to favor cell proliferation, indicating an important role for ion channels in oncogenesis (7). However, the role of PKC in cancer has been controversial. For example, the genetic deletion of PKCα in mice causes them to spontaneously develop colon cancer. Deletion of PKCζ in mice, which are phosphatase and tensin-homolog (PTEN) haplo-insufficient, results in more aggressive prostate tumors (8). In these tumors, it appears that a loss of PKC function enhances oncogenic susceptibility. In contrast, nPKCs appear to function as oncoproteins in several carcinomas. Exogenously enhanced PKCε expression in mouse prostate appears to promote the formation of preneoplastic lesions while PKCε deletion hinders the development and metastasis of prostate cancer (8). PKCε overexpression is associated with a loss of contact inhibition and increased tumorigenicity (9). PKCδ reportedly promotes the progression of pancreatic cancer (8). PKC expression in breast cancer appears to be more complicated and cancer-type specific. Increased PKCδ mRNA is correlated with diminished survival in ErbB2-positive breast cancer as well as estrogen receptor-positive breast cancer. Conversely, increased expression of PKCβ in hormone-insensitive breast cancer is correlated with higher survival rate. Similarly, invasive breast carcinomas express PKCβ(A509V) that causes loss of function (8). While the role of PKCs in cancer is well known, literature regarding their regulation of ion channels and the subsequent modulation of cancer types is still lacking.

PKC enzymes as well as voltage-gated potassium (K_V_) channel currents are found to be dysregulated in Alzheimer’s disease (10, 11). PKC activation was found to rescue premature cell death and improve behavioral outcomes in murine models of Alzheimer’s disease by increasing the processing of amyloid precursor proteins (10).

PKCs also play a role in HIV infections. The HIV gene *nef* is implicated in enhancing disease progression in several ways, one of which is the enhancement of viral replication and virion infectivity. Phosphorylation of Nef by PKCθ and δ at Ser6 of its N terminus is important in the mediation of Nef’s effects on viral replication (12).

The role of PKCs in ion channel regulation has been studied for decades. Although ligand-gated channels are not covered in this review, they are also modulated by PKC. For example, depending upon which channel subunit is phosphorylated by PKC, glycine receptors (GlyR) show enhanced desensitization, increased receptor function, or greater cell surface expression. Phosphorylation at different sites on the γ2 subunit of the gamma-aminobutyric acid-A receptor can cause altered sensitivity to benzodiazepines and ethanol or increased amplitude of inhibitory postsynaptic potentials. Additionally, PKC phosphorylation of the α4 subunit can lead to increased gamma-aminobutyric acid-A surface expression and activity. PKC phosphorylation at different sites on the neuronal nAChR can either lead to enhanced desensitization or increased mature receptor expression at the cell surface (13).

## Identification & validation of PKC sites

### SDS-PAGE gel-based tools

The advantage of these techniques is that they can be applied to cell lysates; however, they are not effective in the isolation of phosphorylated residues. Proteins can be separated using SDS-PAGE gels and stained using fluorescent gel stains that bind to phosphorylated proteins, effectively separating phosphorylated from unphosphorylated proteins (14).

Phosphoantibodies directed at the residue that is phosphorylated (pSer, pThr, or pSer/pThr), serine and/or threonine in the case of PKC, are often used in Western blots to identify phosphorylated proteins.

### Proteomics

Proteomics is the gold standard for the identification of posttranslational protein modifications, including phosphorylation. “Top-down” mass spectrometry (MS) is used to identify the number of modifications to a protein when compared to the pure, unmodified protein, which is used as a control or compared to a calculated mass based on the native protein sequence. For example, in the case of phosphorylation, this enables the detection of multiple phosphorylated residues derived from the mass difference between the calculated theoretical mass and the obtained intact mass of the protein. A second, often complementary, approach is “bottom-up” MS, which involves an in-gel or in-solution digest, usually with trypsin, of target proteins into its component peptides. These peptide fragments are then isolated using tandem MS to identify individually modified (phosphorylated) amino acid residues (15).

Quantitation of phosphorylated protein relative to unphosphorylated protein can be performed using liquid chromatography combined with inductively coupled protein MS by evaluating the abundance of phosphate relative to other elements in the protein sample (16). This is a layer of evidence that goes above and beyond the mere identification of a phosphorylated residue in that it can deduce the proportion of protein that is phosphorylated to lend itself to conclusions regarding the biological effects of a predominantly phosphorylated protein.

### Site-directed mutagenesis

Upon the identification of phosphorylated sites using proteomics, a robust follow-up approach involves making PKC-inert mutants, often Ser/Thr →Ala, and testing channel electrophysiology to confirm the loss of PKC effect on the channel. Similarly, introducing a negative charge at the putative phosphorylation site by introducing a negatively charged amino acid (Ser/Thr →Asp/Glu) as a potential phosphomimetic residue can be an effective way to assess the importance of the [PO_4_]^2-^ moiety in the phosphorylated form of the protein. Our lab has recently developed an optogenetically activated PKCε that can be used for targeted study of protien phosphorylation by this PKC (17).

### PKC modulator compounds

The lack of specific PKC isozyme–activating modalities has significantly impacted the progress of studies pertaining to the role each isozyme plays. Older literature, as is evident from later sections in this review, relied on pan-PKC modulatory compounds. Relatively isozyme-specific small molecules are now available to activate/inhibit some of the PKC enzymes; however, they also have a low affinity for other PKC isozymes, which somewhat limits their utility.

#### Nonselective PKC activators

DAG or its analog 1-oleoyl-2-acetyl-sn-glycerol binds the C1 domain of cPKC and nPKC subtypes resulting in activation. The hydrolysis of DAG releases PKC from its activated conformation and allows it to regain its autoinhibited conformation (8).

Many PKC activator small-molecule compounds are available to activate the PKC enzyme family. PKCs can be activated by phorbol esters, like phorbol 12-myristate 13-acetate (PMA) and prostratin, which bind to their C1 domain in place of DAG (4). Phorbol esters, however, cannot be metabolized by the cell and result in a continuous activation of these kinases causing downregulation of PKC isozymes (8). Bryostatin-1 is a pan-PKC activator that has a low nanomolar affinity for PKC isozymes. It competes with phorbol esters for PKC-binding sites (18). Ingenol 3-angelate is a nonspecific PKC activator that behaves like a partial agonist/antagonist of PKCs and is variable in its ability to activate the different PKC isozymes (19). It is able to recruit novel PKCs to the cell membrane more efficiently than some cPKC isozymes. It does, however, recruit PKCβ to the plasma membrane and is hence not a truly specific activator of nPKC isozymes (16). Thymeleatoxin (TMTX) is similar in principle, in that it is better able to activate the conventional class of PKCs compared to other PKC isozymes (20).

#### Nonselective PKC inhibitors

Staurosporine and bisindolylmaleimide (BIM) are commonly used pan-PKC inhibitors that inhibit PKCs by competing with ATP at its binding site. Both these compounds are nonselective, inhibiting other kinases, like PKA and protein kinase G, besides PKC. Ruboxistaurin (LY333531) and enzastaurin (LY317615) are staurosporine derivatives that are more selective for PKC (21). Both BIM and staurosporine are found to block several ion channels, including cardiac Na_V_, Ca_V_, and K_V_ channels, and are hence nonspecific for ion channel studies. These effects are summarized by Son *et al*. (21). Calphostin C is a more selective pan-PKC inhibitor; however, it requires light for activation (21) and is less commonly used.

## Phosphorylation of ion channels by PKC

**Figure 2:**
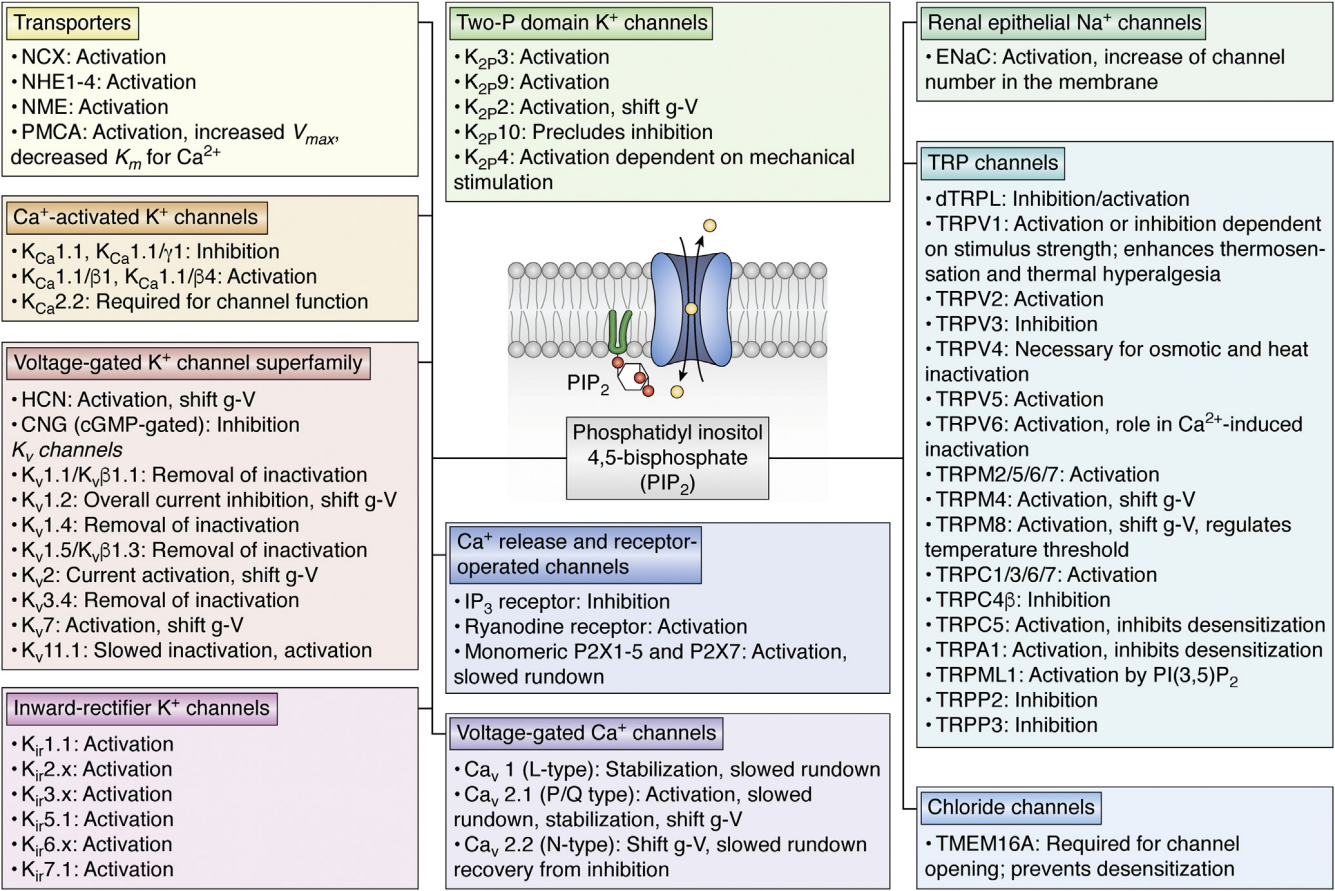
**PIP**_**2**_**is a master regulator of the function of ion channels** (22, 23, 24, 27, 86, 103, 112, 171, 172). Ten different ion transporting membrane protein families are shown to be dependent on PIP_2_ for their activity and the specific direction of the control of the activity of these proteins by PIP_2_ is indicated. PIP_2_, phosphatidylinositol (4,5) bisphosphate.

**Figure 3:**
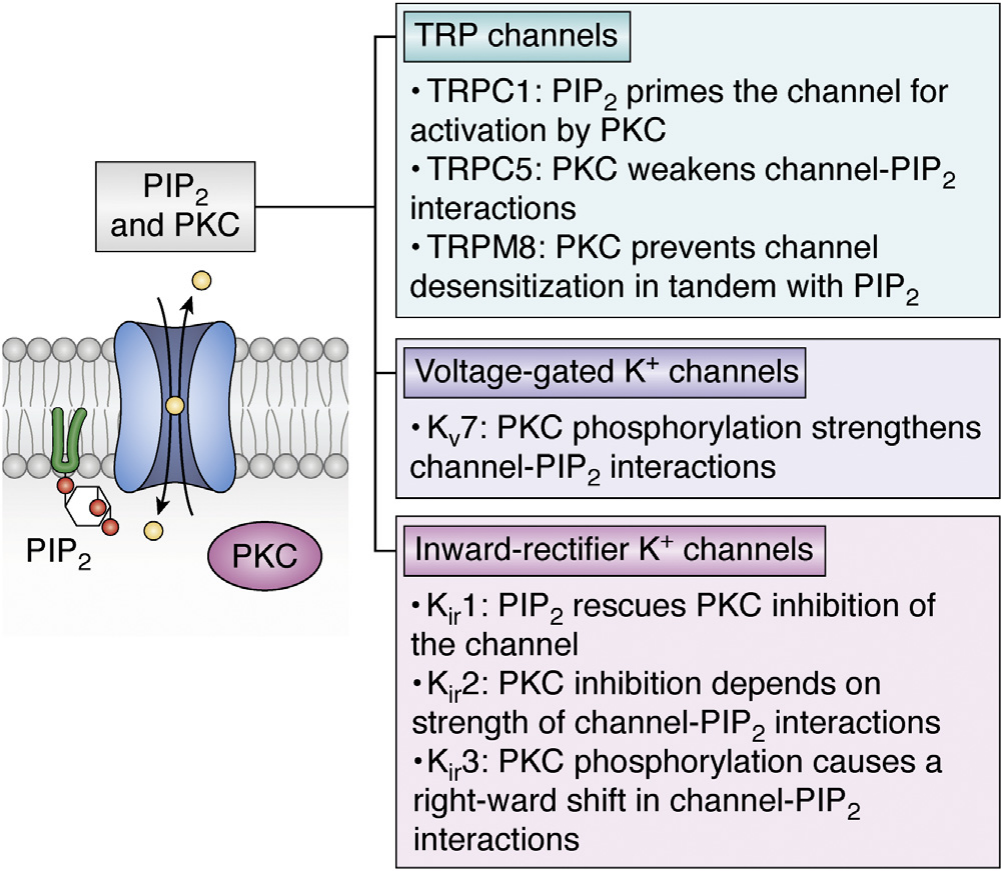
**PKC can regulate the activity of ion channels in a PIP**_**2**_**-dependent manner** (31, 39, 57, 58, 63, 95, 173). Examples of ion channels where PKC regulation of their activity has been linked to alteration of channel–PIP_2_ interactions. PIP_2_, phosphatidylinositol (4,5) bisphosphate.

We have all come to accept protein phosphorylation as a ubiquitously used mechanism to regulate protein activity in response to the action of protein kinases. Ion channel proteins undergo conformational transitions to occlude a water-permeable pathway across the membrane at specific constriction points referred to as “gates.” A stimulus that opens the gates results in an ion-permissive conformation that allows the cell to polarize the membrane and perform a multitude of key cellular functions. Yet, how the posttranslational phosphorylation event couples to the control of the gates has remained elusive. A common thread through the ion channel superfamily is channel regulation by the low abundance membrane phospholipid, PI(4,5)P_2_ or PIP_2_ (22, 23). PIP_2_ is localized near the channel gates and appears to control channel gating mainly *via* electrostatic interactions with key basic residues that allosterically couple to the gates. Multiple posttranslational modifications, including phosphorylation, also couple allosterically to the channel sites that interact with PIP_2_ to modulate gating. This mechanism provides insight as to how allosterically coupled sites in the protein are utilized by nature to regulate the activity of ion channel proteins. Yet, the precise details of the allosteric coupling between phosphorylation sites and channel–PIP_2_ sites remain to be elucidated by structural studies and molecular dynamics (MD) simulations. Reviews have compiled the reported effects of PIP_2_ on various ion channels (22, 23, 24). Figure 2 summarizes our present knowledge of channels regulated by PIP_2_. In addition, we have collated evidence detailing the role of PIP_2_ in the regulation of PKC-mediated channel modulation (Fig. 3). The various isoforms of PKC identify similar but distinct consensus sites to phosphorylate a vast number of proteins, including ion channels. Table 1 lists the most common isoform specific PKC consensus sites utilized by “phosphosite plus” to identify putative PKC sites (25) (Table 1). It is evident from this table that, while these enzymes belong to the same enzyme family, their ability to recognize different amino acid motifs introduces a layer of complex biological regulation upon the proteome. In this review, we examine the hypothesis that PKC regulation of ion channel activity proceeds *via* allosteric modulation of channel–PIP_2_ interactions. The effects of PKC and any intrinsic PIP_2_ regulation thereof, are detailed in subsequent sections pertaining to each ion channel family.

**Table 1:** Commonly observed consensus sites

PKC isoform	Consensus sites	Experimental examples (ref)
α	R(R/X)(***S/T***)X(R/K)	hERG – T74 (PR***T***QR) (99)
βI	(R/K/X)X(***S/T***)XR	
βII	(R/X)X***S***X(R/K)	
γ	XX***S***(K/F/X)(R/K)	
δ	RX***S***(F/X)(R/K)	mTRPC6 – S448 (AA***S***FT) (36)
ε	(R/X)X***S***X(R/K)	NaV1.5 – S1505 (LG***S***KK) (145)
η	(R/E/T)X***S***X(R/X)	
θ	(R/X)X***S***X(R/X)	
ι	XX(***S/T***)(F/K/X)R	
ζ	(R/X)X(***S/T***)(F/X)X	

(www.phosphosite.org—Hornbeck *et al.*, 2015 (25); consensus sites were deduced using Uniprot)

## Transient receptor potential channels

Transient receptor potential (TRP) channels are nonselective cation channels in mammals that are divided into six families TRP canonical (TRPC), TRP melastatin (TRPM), TRP vanilloid (TRPV), TRP ankyrin, TRP polycystic (TRPP), and TRP mucolipin. Structural and functional details have been recently reviewed, see (26). These channels are also strongly regulated by PIP_2_ (24, 27).

### TRPC channels

TRPC channels are divided into four groups based on sequence homology (I) TRPC1; (II) TRPC2; (III) TRPC3, TRPC6, TRPC7; and (IV) TRPC4 and TRPC5. They are structurally similar with six transmembrane helices, a calmodulin/inositol triphosphate–binding region and the TRP motif (amino acids EWKFAR). TRPC channels have been shown to form both heterotetramers and homotetramers (28). TRPCs are activated by G_q_-linked G-protein–coupled receptor and receptor tyrosine kinase signaling.

#### TRPC1

Evidence of PKC phosphorylation of TRPC1 channels is present in the literature (29). PKCα is thought to facilitate the permeability of Ca^2+^ through the channel (29). TRPC1 channels are expressed in vascular smooth muscle cells and function as store-operated Ca^2+^ channels. The nPKC, PKCδ, is highly expressed in rat mesenteric artery musculature and is the PKC isoform presumed to be responsible for the PIP_2_ sensitization of TRPC1 channels in these cells (30). It has been suggested that PKC phosphorylation of TRPC1 channels sensitizes the channel to its ligand; PIP_2_ and TRPC1 activation by dioctanoyl PI(4,5)P_2_ (diC8-PIP_2_) is abolished by PKC inhibitors (31). In fact, TRPC1 channels that were previously found to be activated by PKCδ stimulation using phorbol 12,13-dibutyrate (PdBu) remained inactive when only diC_8_-PIP_2_ was included in the pipette. In the presence of both diC_8_-PIP_2_ in the patch pipette and PdBu, TRPC1 currents were significantly greater than those recorded with either manipulation alone (30). These reports emphasize a role for PIP_2_ in priming the TRPC1 channel for activation by PKCδ. TRPC1 phosphorylation levels increased by activating PKCα activity. Another PKC isoform, PKCα, can promote insulin secretion *via* TRPC1 phosphorylation in INS-1E cells (32).

#### TRPC3, TRPC6, and TRPC7

TRPC3, TRPC6, and TRPC7 are known to be directly activated by DAG (33, 34), independent of PKC. Through site-directed mutagenesis, it was observed that TRPC3 was inhibited by PKC through phosphorylation of a conserved serine, S712. The PMA-induced phosphorylation of the channel was abolished in the mutant S712A, confirming that PKC directly acts on TRPC3 to inhibit channel activity; however, specific isoforms of PKC were not probed in this study (35). Others reported a similar effect of PKC on TRPC6 channels expressed in vascular smooth muscle cells (36). In preliminary experiments, multiple amino acid residues were implicated in the inhibition of TRPC6 by PKC. Subsequently, S448, a predicted consensus site for the nPKC isoform PKCδ, was mutated to alanine, which abolished channel inhibition after PKC activation. Additionally, knocking down PKCδ in a vascular smooth muscle cell line abolishes PKC-mediated channel inhibition and potentiates vasopressin-induced Ca^2+^ entry (36).

#### TRPC4 and TRPC5

PKC phosphorylation of murine TRPC5 at T972 causes desensitization of the channel. This residue is part of a VT^972^TRL motif, and it is likely that PKC phosphorylation at T972 may control the interaction of TRPC5 with PDZ domains of other proteins, such as Na^+^/H^+^ exchanger regulatory factor (NHERF), precluding channel interactions with DAG (37, 38). Using an elegant optogenetic tool to control rapid dephosphorylation of PIP_2_, it was recently shown that PKC-induced desensitization of TRPC5 through Thr972 is caused by weakening channel–PIP_2_ interactions (39). In contrast, DAG enhanced channel–PIP_2_ interactions and activated the channel (39).

### TRPV channels

The TRPV channel family has six members, TRPV1–6. While these channels belong to the same family and share significant sequence homology, they differ in that TRPV1–4 channels are nonselective cation channels while TRPV5 and TRPV6 are highly calcium selective. Additionally, they do not respond to temperature stimuli (26). Regulation of TRPV channels by PIP_2_ has been extensively studied in the literature and is summarized in Figure 2. Most members are positively regulated while some, like TRPV3, are inhibited by PIP_2_ (24).

#### TRPV1

Phosphorylation of TRPV1 channels has been studied extensively (37). This mechanism has garnered attention due to the role of TRPV1 channels as well as PKC in the mediation and enhancement of hyperalgesia after inflammation. The novel isoform, PKCε, is coexpressed with TRPV1 channels in dorsal root ganglia (DRG) neurons. Activation of the neurokinin-1 (NK-1) receptor in DRG neurons initiates the Gq signaling cascade to activate PKCε that modulates TRPV1 channel function, which is thought to play a role in heat hyperalgesia (40). PKCε enhances TRPV1 activity by potentiating the effect of capsaicin on TRPV1 currents. These effects were reported after the use of PMA as well as ATP directly, along with capsaicin (41). Desensitization of TRPV1 currents, following successive applications of the channel agonist, capsaicin, was abolished after the application of PMA, indicating that channel phosphorylation was responsible for making the channel responsive to capsaicin again (42). The abolishment of PKC-mediated regulation of rat TRPV1 channels when S502 and S800 are both mutated to alanine has been described in the literature (41, 43). These PKC phosphorylation sites are further confirmed in that the TRPV1 double mutant does not regain its sensitivity to capsaicin after the application of PMA (42). Joseph *et al*. (44) identified the PKC-phosphorylated residue, S801, in murine TRPV1 to be responsible for inflammation-mediated sensitization of the channel to capsaicin. Further, the mechanism of PKC-mediated activation of TRPV1 channels was elucidated through experiments with the TRPV1(S502A/S800A) double mutant to be due to an increase in the open probability of the channel (45).

While direct evidence is lacking (*e.g.*, through MS), these residues appear to be constitutively phosphorylated in the presence of ATP (41).

#### TRPV3

Heterologously expressed TRPV3 currents in HEK293 cells are found to be enhanced by PMA while this effect is blocked by PKC inhibitors (46). Literature verifying whether this is a direct effect of PKC and the elucidation of the phosphor-acceptor residue/s is lacking.

#### TRPV4

TRPV4 is thought to be involved in the mediation of mechanical hyperalgesia. Activation of human TRPV4 channels is enhanced by PMA, as well as the inflammatory mediator bradykinin, in heterologous expression in HEK293 cells. PKC phosphorylation of the residues S162, T175, and S189 has been implicated in conferring hypersensitivity to the TRPV4 channel. Mutation of these residues to alanine abolished the effect of PKC on TRPV4 channels (47).

#### TRPV5

These channels are found in renal distal convoluted tubules and connecting tubules and regulate Ca^2+^ reabsorption in the kidney. Parathormone also regulates Ca^2+^ by partially acting on this channel. This action is attributed to the increased surface expression of the channel. PKC enhances TRPV5 activity by inhibiting caveolin-mediated TRPV5 downregulation (48).

#### TRPV6

TRPV6 channels are regulated by calmodulin and PKC. PKC-mediated phosphorylation of TRPV6 rescues calmodulin-mediated TRPV6 channel inactivation. Through this mechanism, PKC-mediated phosphorylation acts as a switch to regulate the amount of Ca^2+^ influx through TRPV6 channels (37).

### TRPM channels

Eight subtypes of TRPM channels, TRPM1–8, have been identified. Unique to this family of channels is an N-terminal “TRPM homology region” that is involved in channel trafficking and assembly. These channels are largely Ca^2+^ permeable channels and lack any significant sequence homology (28). Based on structural similarity, they are divided into four groups, (I) TRPM1 & TRPM3; (II) TRPM4 & TRPM5; (III) TRPM2, TRPM6 & TRPM7; and (IV) TRPM8 (28). TRPM channels are sensitive to the presence of PIP_2_ in the membrane. The conserved TRP motif is thought to bind to PIP_2_. Typical effects of PIP_2_ association with the members of this family include increased current, a leftward shift in activation voltage causing the channel to open at more negative voltages and a decrease in PIP_2_ affinity upon mutation of basic residues in the channel (22).

#### TRPM1 & TRPM3

The activation of PKCα potentiates the TRPM1 current in rod-driven optic nerve bipolar cells. TRPM1 current is inhibited by Mg^2+^, and PKCα activation possibly relieves this inhibition (49).

#### TRPM4 & TRPM5

TRPM4 channels require Ca^2+^ for activation. TRPM4 channels are activated by PKC through an enhanced sensitivity to Ca^2+^ (50). In experiments with PMA, there was a left shift in the Ca^2+^ concentration–response relationship measured in HEK cells with the EC_50_ decreasing to 4 μM Ca^2+^ in PMA-treated cells compared to 15 μM in control conditions. The effect of PMA was absent in cells expressing channel mutants S1145A or S1152A, indicating that these are the phosphorylation sites for PKC on TRPM4 (50). Additionally, PKC-dependent activation of TRPM4 channels is essential for cerebral artery vasoconstriction. Enhanced Ca^2+^ influx into cerebral muscle cells is mediated through the activation of voltage-dependent calcium channels Ca_V_ leading to arterial vasoconstriction in a cascade initiated by the PKC-dependent activation of TRPM4 channels (51).

#### TRPM8

TRPM8 channels are highly expressed in the DRG and play an important role in opioid-induced cold hyperalgesia. S1040 and S1041 are two PKC phosphorylation sites in the TRPM8 channel. Mutation of these residues was able to prevent the μ-opioid receptor (MOR)–induced inhibition of TRPM8 desensitization. Activation of MOR by morphine enhances the hyperexcitability of TRPM8-expressing neurons and induces a PKCβ-mediated reduction of TRPM8 desensitization. TRPM8 channels rely on the integrity of their association with the membrane phospholipid PIP_2_, to prevent channel desensitization, that is, PIP_2_ depletion plays a role in channel desensitization. This TRPM8 desensitization was blocked by using a phospholipase C enzyme inhibitor, U73122, as well as the PKCβ inhibitor, enzastaurin. This MOR/PKCβ-dependent modulation of TRPM8 appears to be dependent on the presence of PIP_2_ in the membrane and may underlie the onset of cold hyperalgesia caused by repeated administration of morphine (52).

### TRPP channels

These are Ca^2+^ permeable ion channels. Mutations in TRPP1 or TRPP2 result in renal failure due to autosomal dominant polycystic kidney disease (37). There is a dearth of literature regarding the PKC regulation of these channels.

## Inwardly rectifying potassium channels

Inwardly rectifying potassium (K_ir_) channels are a large family of K^+^ conducting channels that are expressed throughout the body. These channels are inwardly rectifying, in that, they pass larger currents in the inward direction than the outward, and are divided into seven subfamilies (53). Activation of all members in this family is dependent on PIP_2_ (54).

### K_ir_1

K_ir_1.1 was shown to be insensitive to the phorbol ester PMA, suggesting that PKC does not modulate this channel (55). Yet, lack of PMA modulation of K_ir_ currents is not evidence for lack of phosphorylation as has been strongly suggested by studies with K_ir_2 channels and modulation of channel–PIP_2_ interactions (see later under K_ir_2 channels). In fact, phosphorylation of S4 and S201 by PKC is found to be essential for surface expression of K_ir_1.1 (56). It was later shown that PIP_2_ attenuates PKC-mediated channel inhibition (57). Additionally, PKC shifted the pH sensitivity of these channels toward more neutral pH levels *via* a charge–charge interaction (58). This effect of PKC seemingly diminished the sensitivity of the channel to PIP_2_.

### K_ir_2

Scherer *et al*. (20) showed a small but significant inhibition (∼10%) of K_ir_2.1 channel activity using PMA. Eighty percent of the activity of K_ir_2.2 is inhibited by PMA (20). In order to identify the specific PKC isoforms responsible for the inhibition of K_ir_2.2, Scherer *et al*. (20) used TMTX to activate the conventional isoforms of PKC (α, β, and γ) and ingenol to activate the nPKC isoforms (δ and ε). TMTX treatment resulted in an 80% current inhibition similar to PMA, while ingenol had no effect implicating a cPKC isoform. The application of a PKCβ inhibitor, (3-(1-(3-imidazol-1-ylpropyl)-1H-indol-3-yl)-4-anilino-1H-pyrrole-2,5-dione, to TMTX-treated cardiomyocytes rescued the PKC-mediated inhibition of K_ir_2.2 activity, clearly demonstrating an important role for PKCβ in diminishing I_K1_ currents, which are the K_ir_2-mediated currents in the heart. The I_K1_ current decrease was linked to an increased risk for arrhythmia (59), and the differential regulation of K_ir_2.1 and K_ir_2.2 by PKC in the heart is a druggable target in conditions like Andersen’s syndrome, which is associated with impaired PIP_2_ regulation of K_ir_2.1.

K_ir_2.3 activity was robustly inhibited upon application of PMA in oocyte experiments, indicating that PKC likely phosphorylates this channel (20). A combination of chimeras and site-directed mutagenesis was used to circumvent the possibility of nonfunctional channels in order to identify the PKC phosphorylation site on K_ir_2.3 (60). PKC consensus sites S5, S36, S39, and T53 located on the N terminus of K_ir_2.3 were evaluated. Of these, the absence of T53 rendered the channel insensitive to PMA. Further, Du *et al*. (61) used a mutant K_ir_2.3(I213L), which increases the apparent affinity of the channel for PIP_2_. This mutant was refractory to the effects of PMA suggesting that the effect of PKC on K_ir_2.3 is governed by channel–PIP_2_ interactions. These authors used this hypothesis to develop and test a mutant of K_ir_2.1—(R312Q)—which has a decreased affinity for PIP_2_ than the native channel. Close to 50% of the activity of K_ir_2.1(R312Q) was inhibited by PMA, further supporting the idea that decreased channel–PIP_2_ affinity is paramount to conferring PKC sensitivity to the channel (61).

### K_ir_3

**Figure 4:**
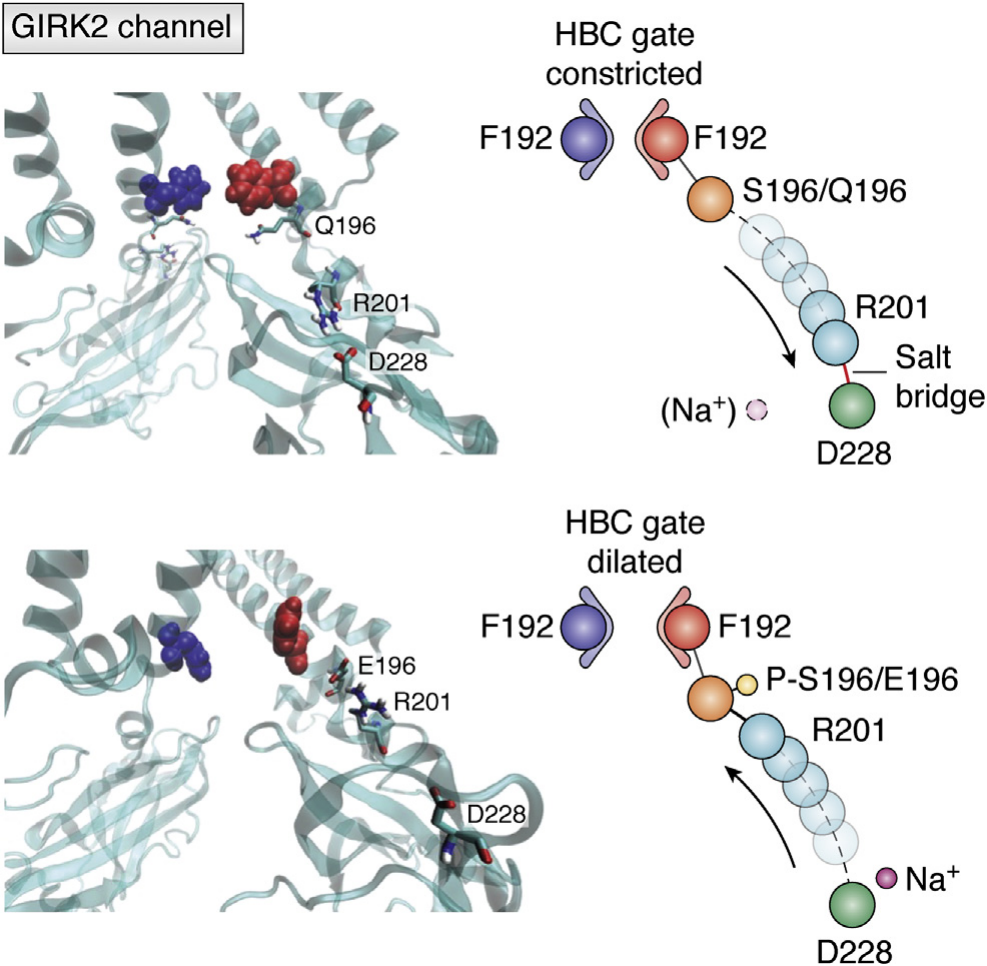
**D228 and phosphorylated S196 compete for R201 to gate the GIRK2 channel** (55). In the absence of Na^+^ and phosphorylation of S196, the D228 residue (that can bind Na^+^) forms a salt bridge interaction with R201 stabilizing the helix bundle crossing (HBC) gate of the GIRK2 channel in the constricted conformation (*top*). Phosphorylation of S196 competes away R201 from D228 causing HBC channel gate dilation (*bottom*). GIRK, G-protein-gated inwardly rectifying K^+^ channel.

The K_ir_3 family of ion channels is also known as G-protein-gated inwardly rectifying K^+^ channels (GIRKs). Studies describing the simultaneous effect of PIP_2_ depletion and PKC activation through GqPCR signaling have shown that both of these factors profoundly inhibit GIRK channel activity in heterologous systems (62, 63). Heteromeric GIRK1/4 channels were inhibited by PKC through activation by PMA in heterologous systems (62). This inhibition occurred through the suppression of channel open probability rather than suppression of channel conductance. Through site-directed mutagenesis, these authors attempted to evaluate the PKC phosphorylation sites on GIRK1 & GIRK4. They showed that GIRK1(S185) and GIRK4(S191) are amino acid residues that appear to be essential for the effect of PKC on channel activity. The GIRK2 atomic resolution structure with PIP_2_ has been determined (64), allowing MD simulations to assess the mechanism by which phosphorylation of a particular site of this channel may control gating. Adney *et al.* (65) identified a triad of interacting residues critical in GIRK2 channel gating: R201 interacts electrostatically with either phosphorylated S196 (P-S196) (stabilizing the helix bundle–crossing gate in the dilated state) or with D228, the Na^+^-binding site (stabilizing the helix bundle–crossing gate in the constricted state that could be reversed by Na^+^ binding co-ordinated by D228) (Fig. 4). S196 is the putative PKC GIRK2 residue corresponding to the GIRK1(S185) and GIRK4(S191). The P-S196–R201 interaction allosterically decreased PKC sensitivity in a PIP_2_-independent manner, while disruption of this electrostatic interaction by mutation of either residue modulated PKC sensitivity. These authors concluded that Ser-196 exerts allosteric control over PKC inhibition of Kir3.2, rather than being itself a direct target of PKC phosphorylation. Thus, the role of the Ser residue at this position is more complex than previously thought. Figure 4 highlights that such a mechanistic insight, clarifying a previous erroneous interpretation that Ser-196 served as the direct target of PKC phosphorylation, could only be achieved by the availability of the GIRK2 structure and the MD simulation analysis able to probe the mechanism (55). Zhang *et al*. (66) tested mutant GIRK4 constructs where Thr37, Thr67, Thr70, Ser209, and Ser233 were each mutated to alanine and treated with PMA. They observed a PMA-mediated current inhibition in all the mutant channels. They also tested a pentamutant with all five sites mutated to alanine and observed a similar inhibition upon PMA treatment. These reports suggest that PKC enzymes likely phosphorylate sites distinct from those tested by Zhang *et al*. (48). Given that PKC has several distinct isoforms, these findings remain to be further explored in order to elucidate specific PKC isoform effects.

PKC isoform-specific regulation of cardiac GIRK4 containing heteromers has been described in the literature (63, 67). cPKC isoforms, PKCα, PKCβI, and PKCβII, reportedly inhibit I_KACh_ mediated through GIRK1/4 channels, while nPKC isoforms, PKCε and PKCδ, appear to enhance I_KACh_ (67).

Channel–PIP_2_ interactions appear to govern the effect of PKCδ on Kir3 channels. Exogenous application of diC8 to oocytes that express PKCδ causes a right shift in the dose–response relationship of the channel with diC8-PIP_2_ (63). GIRK2 channels have also been shown to be inhibited by PKC (65), however, PKC isoform-specific studies are lacking.

### K_ir_6

K_ir_6 channels are also known as K_ATP_ channels, as this potassium channel subfamily is inhibited by ATP. Four K_ir_6.*x* subunits along with four regulatory sulfonylurea receptor subunits (SURs) form an octameric channel complex (68, 69, 70).

Kir6.1/SUR2B is inhibited by PKC through phosphorylation on the pore-forming K_ir_6.1 subunit. PKCε is implicated in the regulation of K_ir_6.1 (71).

The cardiac channel, Kir6.2/SUR2A, and the pancreatic channel assembly, Kir6.2/SUR1, are both activated by PKC. In addition, phosphorylation also increases the open probability of the channel. The mechanism leading to an increase in channel activity has been suggested to rely on decreasing the positive co-operativity of ATP at these channels, hence diminishing the inhibitory effect of ATP. The effects of PKC are seen even in the absence of the SUR subunits in experiments with the Kir6.2ΔC26 mutant channel. In support of a direct effect of PKC through Kir6.2 phosphorylation, mutation of the putative phosphorylation site, Thr180, abolished the stimulatory effects of PKC on the channel (72).

### K_ir_7.1

PKC inhibits the activity of K_ir_7.1 channels by phosphorylating S201 on the C terminus of the channel (73). Comparable inhibition of channel activity was seen when either the pan PKC activator, OAG, or TMTX, which predominantly activates cPKC isoforms (74), was applied to the channel.

This indicates that cPKC isoforms are likely implicated in K_ir_7.1 channel inhibition. K_ir_7.1 has been shown to be expressed in several tissues including the basolateral membrane of renal nephrons (75). These channels are thought to couple to the Na^+^/K^+^-ATPase pump and participate in the regulation of plasma K^+^ (76).

## K_V_ channels

K_V_ channels are a highly conserved group made up of 12 subfamilies that house 40 K_V_ channels. K_V_ channels are widely expressed throughout the body especially in tissues that display electrical activity like the heart, skeletal muscle, and the brain (77). The K_V_α subunits form homomeric or heteromeric co-assemblies to form functional protein channels. Additional diversity in the channels is offered by the heteromerization of K_V_α subunits with β subunits (78).

### K_V_1

K_V_1.4, a member of the *Shaker* Kv-channel family, was inhibited by PKC isozymes in different cell systems. Reduced membrane expression is implicated in the decrease in current amplitude due to PKC-mediated protein downregulation (79).

### K_V_1.5

K_V_1.5 channels are responsible for the ultra-rapid potassium channel current mainly seen in the atria of the heart (K_UR_). There is a corpus of literature that describes the inhibition of K_V_1.5 current *via* Gq receptor signaling. These effects are ascribed to PKC activation by the second messenger, DAG. For example, angiotensin II acting on AT_1_ receptors, endothelin-1 acting on ET_A_ receptors, thromboxane A_2_ acting on TP receptors, and serotonin acting on 5HT_2A_ receptors, all cause K_V_1.5 channel inhibition through PKC activation downstream of their Gq-signaling pathways (80, 81). This decrease in current was assessed to be due to reduced membrane expression through increased endocytosis and protein downregulation as examined in immortalized atrial cells (82). The novel isoenzyme, PKCε, was involved in angiotensin II inhibition of mesenteric K_V_ channels through PKCε, while U46619, a thromboxane A_2_ analog, inhibited rat pulmonary artery K_V_ channels through PKCζ (83, 84). Co-expression of a β subunit, K_V_β1.2, was suggested to enhance the effect of PKC on I_KUR_ (85). PIP_2_ has been shown to exert a dual regulation on the activity of K_V_1.2 channels, similar to that reported for Ca_V_ channels (86). This dual regulation is characterized by PIP_2_ stabilization of channel activity by slowing rundown as well as causing a right shift in the voltage-dependent activation of these channels. PIP_2_-mediated inhibition of the fast inactivation properties of these channels have also been reported (86). Yet, no information is available on PKC-dependent effects on K_V_1 channels proceeding *via* modulation of channel–PIP_2_ interactions.

### K_V_2.1

Exogenous application of PIP_2_ appears to protect the Kv2.1 channel from current rundown while PIP_2_ depletion facilitates it. Additionally, PIP_2_ depletion enhances the rate of channel inactivation (87).

### K_V_3

This subtype of K_V_ channels is an A-type potassium channel that displays fast inactivation. Covarrubias *et al*. (88) reported PKC-mediated inhibition of fast inactivation through the phosphorylation of serine residues on the N-terminal end of K_V_3.4. In a comprehensive study, these authors used purified brain PKC along with a peptide resembling the N-terminal region of K_V_3.4 to show phosphorylation of the N-terminal peptide *in vitro*. Subsequent mutation of S15A and S21A, both located in the N-terminal region of K_V_3.4, abrogated up to 50% of the effect of PKC on fast inactivation. These experiments showed that these residues on K_V_3.4 are phosphorylated by PKC and are necessary for the effects of PKC but not sufficient, since a double mutant—S15A/S21A—accounted for 70% of the effect of PKC, indicating that other serine/threonine residues might also be involved. The proposed mechanism of PKC-mediated slow inactivation of K_V_3.4 was evaluated by introducing a phosphomimetic residue, aspartate, at S15. The K_V_3.4(S15D) mutant inactivated significantly more slowly than the WT channel, showing that the negatively charged aspartate residue in place of the serine interfered with critical interactions between the inactivation gate and its receptor site by shielding critical positive charges implicated in fast inactivation and delaying inactivation of K_V_3.4.

### K_V_4

The C terminus of Kv4.2 is phosphorylated by PKC at Ser447 and Ser537. Activation of PKC resulted in phosphorylation at Ser537 in the hippocampus. An increase in the surface expression of phosphorylated K_V_4.2 was observed compared with WT Kv4.2. Additionally, by virtue of the location of these phosphosites in an extracellular signal-regulated kinase (ERK)–recognition motif, the ERK-mediated phosphorylation of these channels is also enhanced (89).

Kv4.3 channels, responsible for outward cardiac *I*_to_ currents during early repolarization, are inhibited by PKCα (90).

### K_V_7

Current through Kv channels or KCNQ channels is known as the M-current. The relationship of these channels with PIP_2_ is well documented in the literature. The depletion of PIP_2_ through Gq activation has an appreciable inhibitory effect on channel activity; however, the PKC component of Gq signaling is also reported to play a role in channel activation (91).

K_V_7.1 or KCNQ1/KCNE1 channels are responsible for the I_Ks_ current that plays a major role in cardiac repolarization. This activity of the cloned human channel was enhanced by the cPKCβII and the nPKCε in heterologous systems. Subtypes other than the cPKCβII and nPKCε have not been reported to modulate the activity of this channel (92). The membrane expression of KCNQ1/KCNE1 channels in rat ventricular cardiomyocytes is decreased through the actions of PKCβII, a cPKC isozyme, after GqPCR activation (93). A decrease in I_Ks_ is known to be a contributor to long QT syndrome (LQTS) (94). Several mutations in KCNQ1 channels have been linked to LQTS, some of which are close to or directly involved in channel–PIP_2_ interactions (95).

As a component of GqPCR signaling, PKC increases channel activity subsequent to early channel inhibition due to the depletion of PIP_2_. PKC-mediated activation of the mutants KCNQ1(R174C) and KCNQ1(R243C) was diminished when the muscarinic M_1_ receptor was used to activate the Gq-signaling pathway. The activation by PKC was enhanced in the KCNQ1(R366Q) mutant channel. Upon probing the mechanism of these effects, it was found that PKC phosphorylation strengthens channel–PIP_2_ interactions to activate the channel; however, these particular LQT1 mutations that show diminished activity are aberrantly regulated by Gq receptor stimulation (95).

In contrast to a slight shift in the activation voltage of KCNQ1, the application of PMA to activate PKC caused a significant rightward shift in the conductance–voltage relationship of K_V_7.2, K_V_7.3, and K_V_7.4. This shift was attenuated in the presence of a PKC inhibitor, chelerythrine (91). These authors were able to recapitulate the effects of phosphorylation by introducing aspartate residues in place of putative phosphorylation sites in the cytoplasmic loop of Kv7.2. S541D, S563D, and S570D mutant Kv7.2 channels, all of which showed a positive shift in their conductance–voltage relationships; however, PMA showed an additional effect, indicating that there may be multiple PKC phosphorylation sites on this channel, possibly due to various PKC isoforms. Additionally, phosphorylation of K_V_7.2 at Ser541 by PKC enhanced or sensitized the channel to the inhibition of currents in response to muscarinic agonists. It thus followed that an S541A mutant yielded greater currents compared to the phosphosite-containing WT channel. Due to its location in the calmodulin-binding segment of the channel, it was observed that PKC-mediated phosphorylation at this residue disrupted calmodulin binding to the channel. Owing to the importance of calmodulin in mediating channel–PIP_2_ interactions, it was observed that PKC phosphorylation of the channels decreased channel–PIP_2_ interactions (96). By comparison in K_V_7.1, a competition of PIP_2_ and the calcified (N-lobe) of calmodulin through residues K526 and K527 was shown to stabilize the channel open state (97).

These channels are sensitive to the presence of PIP_2_ in the membrane and have been extensively reported to be activated by this phospholipid. In the presence of wortmannin to block PIP_2_ synthesis by PI4K, the inhibition of Kv7.2 or KCNQ2 channel currents was significantly greater (22). These data suggested that not only does Kv7.2 retain its dependence on PIP_2_ after PKC phosphorylation but also that the PKC inhibition of the channel itself depends on the PIP_2_ levels in the membrane. In other words, PIP_2_ diminishes the detrimental effect of PKC on Kv7.2 activity (91).

### K_V_11

K_V_11.1 channels or human ether-à-go-go–related gene (hERG) channels are responsible for the rapidly activating delayed rectifier potassium current (I_Kr_) in the heart. Loss of function mutations in hERG channels have been implicated in the development of LQTS, which gives rise to fatal cardiac arrhythmias (98). These channels are regulated by PIP_2_. PIP_2_ activates hERG channels by increasing channel current and attenuating channel run down (99).

hERG channels were found to be sensitive to the action of the PKC-activating phorbol ester, PMA. PMA caused a decrease in hERG-mediated current and an increase in hERG protein expression (98). PKCε inhibited I_Kr_ downstream of AT_1_ angiotensin receptors (100). A channel mutant lacking several residues on the N-terminal end (residues 2 through 354) was found to be insensitive to the inhibitory effect of PMA but remained susceptible to the enhancement in protein expression, showing a direct effect of PKC regulation on hERG channel function (98). PKCα inhibited hERG channels downstream of α_1A_ adrenoreceptor function likely through phosphorylation of T74 on its N terminus (100). The evidence regarding the role of PIP_2_ in PKC regulation of hERG channels is lacking and warrants investigation.

## Two-pore domain potassium channels

Two-pore domain potassium (K_2P_) channels mediate K^+^ background currents that modulate the membrane potential of excitable cells. These channels are heavily involved in pain pathways (101). PIP_2_ induces a left-shift in the voltage dependence of K_2P_2 channels, resulting in increased current through the channel (102, 103). Previously known as the TWIK-related spinal cord K^+^ channel, mutations in K_2P_18 have recently been implicated in familial migraine (101). The K_2P_18.1 current was activated by PMA treatment in experiments using *Xenopus* oocytes (104) suggesting modulation of the channel by PKCs.

## Calcium-activated potassium channels

The calcium-activated potassium (K_Ca_) channel family consists of four subfamilies: K_Ca_1.1 or big K^+^ conductance (BK), K_Ca_2.x or small K^+^ conductance (SK), K_Ca_3.x or intermediate K^+^ conductance, and K_Ca_4.1 (105). K_Ca_1.x channels in rat mesenteric artery smooth muscle cells are inhibited by PKC (106). BK channels have been reported to be PIP_2_ dependent (107, 108); however, a link between PKC-dependent BK channel inhibition and PIP_2_ has not been reported.

SK or K_Ca_2.x channels are gated by changes in the levels of cellular calcium. Three members of this family exist; K_Ca_2.1, K_Ca_2.2, and K_Ca_2.3 (109). Calmodulin serves as the Ca^2+^-gating subunit, while protein kinase CK2 and protein phosphatase 2A modulate Ca^2+^ sensitivity (110). PKC is shown to stimulate SK channels in the cortical collecting duct of rat kidneys by increasing channel open probability (111). K_Ca_2.2 was shown to be PIP_2_ dependent, while phosphorylation of T79 of calmodulin by CK2 inhibited activity by reducing affinity to PIP_2_ (112). A link between PKC-dependent SK channel activation and PIP_2_ has not been reported.

## Voltage-gated calcium channels

These channels are hetero-oligomeric assemblies composed of the pore forming α_1_ as well as β, γ, and α_2_-δ subunits. The activity of voltage-gated calcium (Ca_V_) channels is dually regulated by PIP_2_, in that, it confers stabilization, as depletion of PIP_2_ induces current run down but simultaneously inhibits voltage-dependent activation as it shifts the conductance-voltage (G-V) curve to more depolarized voltages (22, 86). The primary mechanism of Ca_V_ channel regulation is thought to be through phosphorylation (113). Evidence suggesting that Ca^2+^ channels are regulated by PKC abounds in the literature. PKC appears to preferentially phosphorylate the α_1_ subunit of this oligomer, however, phosphorylation of the β subunit has also been reported (69). Links between PKC regulation of Ca_V_ channel activity and PIP_2_ remain to be explored.

### Ca_V_1.x

The activity of rat cardiac Ca_V_ channels expressed in *Xenopus* oocytes is enhanced by Ca^2+^-sensitive PKC isoforms followed by a slight decline in current amplitude (114). Ca_V_ channels in rabbit skeletal muscle are positively modulated by PKC and demonstrate greater Ca^2+^ current amplitude after PKC phosphorylation (115).

PKC isozymes are heavily involved in cardiac cell growth and differentiation, signal transduction, and hypertrophy (4). PKCs are implicated in cardiac ischemia, heart failure, and several other cardiac pathologies. At the tissue level, the rate of spontaneous contraction of rat ventricular cells was significantly increased by almost 86% under the influence of a PKC activator. A concurrent 46% decrease in the amplitude of contraction was also reported (116). These positive chronotropic and negative ionotropic effects were thought to be mediated through the effect of PKC on Ca_V_1.2. PKC activation caused an increase in both the transient and steady-state components of I_Ca_ without altering the reversal potential. The cardiac isoform of Ca_V_ channels in the rabbit heart, Ca_V_1.2, composed of α_1C_, β_2A_, and α_2_-δ_1_ subunits, was sensitive to modulation by PKC. The activity of the long N-terminus α_1C_-containing Ca_V_1.2 channels was enhanced by PKC and blocked by PKC inhibitors like BIM and staurosporine, while the opposite was reported for the short N terminus splice variant (117). This effect was hence deemed to be dependent on the initial segment of the N terminus. The nPKC isozyme, PKCε, was shown to inhibit calcium currents or I_Ca,L_ through Ca_V_1.2 in rat ventricular myocytes, using the specific PKCε activator, εV1–7 (118). The cPKC isozymes also inhibited cardiac I_Ca,L_ (69).

In heterologous systems, rabbit Ca_V_1.2 was inhibited by PKC with a decrease in peak currents, while voltage sensitivity of the channel remained unchanged, alluding to possible differential regulation of PKC based on the specific PKC isozymes expressed in the chosen heterologous system (119). These authors were able to identify two threonine residues on the N-terminal region of the channel, T27 and T31, both of which were necessary for PKC to exert its inhibitory effect on the channel through the negatively charged phosphate groups.

The activity of Ca_V_ channels in the rat retinal pigment epithelium is enhanced by PKC, appearing to be a constitutive posttranslational modification, as is evidenced by the abolishment of its effects by PKC inhibitors but by no change in the presence of PKC activators (120). PKC modulation of this channel in turn regulates the influence of protein tyrosine kinases on the retinal Ca_V_ isoform.

### Ca_V_2.x

PIP_2_ interacts extensively with the voltage sensor domain II (VSD_II_) of the human Ca_V_2.2 channels in the “down” state of this VSD. While the functional state of the channel is undetermined, given that VSD_II_ alone is in the down state, the interaction of PIP_2_ with hCa_V_2.2 at the interface of VSD_II_ and the pore domain (121) provides prevailing evidence of its significant role in Ca_V_2.2 gating. Rat neuronal Ca_V_2.2 and Ca_V_2.3 channel currents were enhanced by PKC through PMA recruitment of DAG-sensitive PKCs in *Xenopus* oocytes, while Ca_V_2.1 and Ca_V_1.2 were insensitive to the effects of PMA (122). These authors found that increase in Ca_V_2.3 activity by PMA was abrogated in the presence of BAPTA (1,2-bis(*o*-aminophenoxy)ethane-*N*,*N*,*N′*,*N′*-tetraacetic acid), a Ca^2+^ chelator, indicating that the effects of PMA were likely mediated through the activation of a Ca^2+^-sensitive PKC (122). However, the effects of PMA on channel activity were only observed if a β subunit was coexpressed. PKC also has a significant effect on the current–voltage relationship and the activation/inactivation kinetics of Ca_V_2.2 and Ca_V_2.3. The current–voltage relation for Ca_V_2.3 showed a significant rightward shift causing the channel to open at more positive voltages, while that for Ca_V_2.2 was left-shifted after PMA treatment. Additionally, the voltage dependence of inactivation for both channels was shifted to the right. PKC phosphorylation also appears to affect the rate of activation and inactivation of Ca_V_2.3 while the rates (τ) of activation and inactivation of Ca_V_2.2 remained close to those of the unmodified channel. Using chimeras, Stea *et al*. (122) further deduced that the I-II linker of the Ca^2+^ channel α_1_ subunit is integral to PKC-mediated channel modulation.

Certain presynaptic, neuronal Ca^2+^ channels contain synaptic protein interaction (synprint) sites that are essential to neurotransmitter release by providing structural elements employed by the vesicle-docking apparatus. The rat Ca_V_2.2 subunit of presynaptic, neuronal channels has a synprint site within residues 718 to 963 in the intracellular loop II-III that interacts with syntaxin and SNAP-25 (123). A phosphorylation site for PKC resides on this intracellular loop within residues 718 to 859. PKC phosphorylation of the α_1B_ subunit prevented the binding of syntaxin 1A and SNAP-25 proteins to the Ca_V_ subunit, which in turn regulated neurotransmission (123). High-voltage threshold Ca^2+^ currents in rat DRG were reportedly enhanced in whole-cell patch-clamp experiments when the pipette internal solution contained constitutively active PKC (124).

## Chloride channels

Chloride channels are involved in the regulation of neuronal excitability, cell volume regulation, salt transport, and apoptosis (125). The members of this family share only functional similarity in that they are chloride channels but are structurally dissimilar.

### Cystic fibrosis transmembrane conductance regulator

The cystic fibrosis transmembrane conductance regulator (CFTR) is an ATP-gated ion channel that allows the passive conductance of chloride ions (126). Mutations in this channel are the causative defect in patients with cystic fibrosis (127). A direct phosphorylation of this channel by PKC was suspected, since PKC consensus sites are conserved across species. Additionally, after the deletion of nine PKC consensus sites from CFTR, PKC regulation of this channel was abolished. Known as the 9CA mutant, this construct is mutated at T582A/T604A/S641A/T682A/S686A/S707A/S790A/T791A/S809A (128). PKC phosphorylation of this channel was reported on residues 686 and 790 in the regulatory domain of the channel (127, 129). The effects of PKC phosphorylation on this channel are that of subtle activation. The cardiac isoform of the CFTR channel was also activated by PKC (130). The more substantial effect of PKC on CFTR appeared to be that of sensitization of the channel to PKA phosphorylation, which activated the channel through its action on several residues. It was hypothesized that PKC phosphorylation exposed previously inaccessible PKA consensus sites for phosphorylation (131). Isozyme-specific studies using a PKCε antisense oligonucleotide diminished the epinephrine-stimulated activation of CFTR, indicating that this nPKC is responsible for sensitizing the channel to PKA (132). PIP_2_ is reported to enable ATP opening of CFTR independently of channel phosphorylation (133). However, this work has not been further elaborated on and the PIP_2_ dependence of CFTR is poorly understood.

### Voltage-gated chloride channels

These channels with 12 membrane spanning domains are crucial in the regulation of cell volume especially in the heart (134). PKC activation through the application of 4β-PMA inhibited the current amplitude of chloride channel (ClC)-1 in HEK-293 cells. Additionally, current deactivation was slower and remained incomplete (135). ClC-3 channels mediate I_Cl.vol_ in cardiac cells (136). Studies with ClC-3 heterologously expressed in oocytes demonstrate the ability of PKC to inhibit channel currents (134). In cardiac myocytes, ClC-3 channels were closed after PKC activation and opened after PKC inhibition or cell swelling. Ser51 in the amino terminus of the ClC-3 channel appears to be the phosphor-acceptor residue for PKC that plays a role in the volume-sensing ability of the channel (136). Not much is known regarding PIP_2_ regulation of these channels.

### Volume regulated chloride channels

Under conditions of hypotonic stress, volume-regulated chloride channel currents are activated by PKCα due to the translocation of this cPKC subtype to the cell surface in cervical carcinoma cells (137). Volume-regulated Cl^-^ currents in guinea pig detrusor smooth muscle cells have been shown to be stimulated by intracellular PIP_2_ (138). No links have been made yet between PKC regulation of these channels and PIP_2_.

### Ca^2+^-activated chloride channels

Although Ca^2+^-activated chloride channels (CaCCs) are probably best known for driving fluid secretion across mammalian epithelia, they are intimately involved in multiple physiological functions in all eukaryotes. CaCCs regulate functions as diverse as smooth muscle contraction, nociception, neuronal excitability, insulin secretion, and cell proliferation and migration in mammals (139). While there are several types of CaCCs, the so-called classical CaCCs are encoded by the ANO1 (TMEM16A) and ANO2 (TMEM16B) genes. They require Ca^2+^ for activation and their activity is stimulated by PIP_2_ (140). PKCα has been shown to stimulate TMEM16A-mediated Cl^-^ secretion in human biliary cells (141). Potential links between PKC regulation of TMEM16A currents and PIP_2_ are awaiting further studies.

## Connexin channels

### Cx43

Connexin hemichannels are proteins that form gap junctions integral to intercellular communication. The hemichannels in adjacent cells join to form a connexon creating a pathway permitting the movement of small solutes and ions. These hemichannels have a tissue-specific distribution. The cardiac subtype includes expression of connexin-43 (Cx43). Fibroblast growth factor-2 (FGF-2) decreases the permeability of cardiac gap junctions by increasing the phosphorylation state of the channel (142). FGF-2 activates receptor cascades linked to PKC activation. PKC abolishes the permeability of connexin 43 gap-junctional channels and hemichannels to large hydrophilic solutes but allows the passage of small, inorganic ions (143). This effect is due to the channel’s phosphorylation of S368, which causes a conformational change leading to altered channel permeability (144).

Doble *et al*. (142) reported that the colocalization of the nPKC isoform, PKCε, with the Cx43 channels at gap junctions on the cell surface was enhanced after FGF-2 activation. PKCε was also found to directly phosphorylate this channel in the human heart (145). Cx43 colocalized and coimmunoprecipitated with PKCα and PKCε in failing and nonfailing myocardium. While PKCα did not appear to directly phosphorylate this channel, its elevation in the failing heart (145) and proximity to areas of Cx43 expression did leave room for further investigation in cardiac malfunction. Cx43 has been shown to depend on intact PIP_2_ for its activity and PIP_2_ hydrolysis triggered by G protein–coupled receptor signaling has been shown to inhibit activity as well as cell to cell communication (146). Yet, the link between specific PKC isoform effects and PIP_2_ remain unexplored.

## Voltage-gated sodium channels

Several phosphorylation sites have been identified on voltage-gated sodium (Na_V_) channels, some of which have yet to be ascribed specific functions (147). No PIP_2_ regulation has been described for Na_V_ channels, thus it is not known whether these channels are an exception to being regulated by PIP_2_ or simply that they have not been studied adequately yet for this form of regulation.

### Na_v_1.2

PKCε has been implicated in the negative modulation of Na_V_1.2 channels by decreasing channel current and slowing its inactivation in hippocampal neurons (147). PKC decreased peak sodium current up to 80% and slowed channel inactivation in rat brain neurons (148). Phosphorylation of a single residue, serine 1506, in the conserved intracellular loop between domains III and IV involved in inactivation of the sodium channel, was required for both modulatory effects (149). Two sites in the I-II linker, S576 and S610, could also be phosphorylated by PKC, and their subsequent effect was to slow the inactivation of this sodium channel while also participating in crosstalk with PKA phosphorylation of the channel (147). PKC phosphorylation appeared to protect the channel from dephosphorylation of PKA sites by phosphatases like calcineurin and PP2A (147).

### Na_V_1.3

The activity of this Na_V_ channel was modulated by PKC in heterologous systems through an increase in current amplitude as well as a left-ward shift in the voltage dependence of channel activation. Both these changes allowed enhanced sensitivity of DRG neurons to nociceptive stimuli (150).

### Na_V_1.4

PKC inhibited the activity of Na_V_1.4 channels as well as slowed channel inactivation (147, 151, 152). An important role for the Ca^2+^-sensitive PKCα has been reported in halothane-mediated inhibition of this skeletal muscle isoform of sodium channels. At low concentrations, halothane could inhibit Na_V_1.4 only when PKCα was coexpressed, resulting in a decrease in current amplitude as well as faster inactivation. The PKC-mediated effect of halothane on channel inactivation was dependent on a conserved phosphorylation site, S1321, in the inactivation gate while the effect on current amplitude was retained in Na_V_1.4(S1321A) channels (153).

### Na_V_1.5

The cardiac isoform of voltage-gated sodium channels, Na_V_1.5, is responsible for the persistent I_Na_ seen in several disease-causing mutations. PKC phosphorylation of this channel diminished persistent I_Na_, appearing to be protective in these conditions (147).

Eleven phosphorylation sites have been identified on Na_V_1.5 through mass spectrometric analyses of murine ventricular tissue (154). One among these, S1505, located in the inactivation gate, was the substrate for PKCε phosphorylation of cardiac sodium channels. PKCε phosphorylation decreased peak Na_V_1.5 currents (147). Additionally, the activation of Ca^2+^ sensitive, cPKC isoforms in the presence of reactive oxygen species inhibited the membrane expression and trafficking of Na_V_1.5 channels (155). This phenomenon was believed to underlie the decreased channel trafficking as well as current amplitude of Na_V_1.5 seen in glycerol 3-phosphate dehydrogenase 1-like (GPD1L) mutations associated with the Brugada syndrome and sudden infant death syndrome (156).

### Na_V_1.6

There are reports that Na_V_1.6 channel currents were inhibited by PKC; however, this reduction was minimal compared to other sodium channels like Na_V_1.2 (147, 157). Specific phosphorylation site information for this channel is lacking as is information regarding its response to individual PKC subtypes.

### Na_V_1.7

Reports in the literature that investigated the role of PKC in Na_V_1.7 regulation have revealed a depolarizing shift in channel activity due to the action of PKCε and the conventional isoform, PKCβII. Additionally, PKC mediated an increase in Na_V_1.7 activity on the background of diabetic neuropathy (147). Another facet of Na_V_1.7 was the ability to generate resurgent currents during the repolarization phase of action potentials. These currents were thought to be important in providing a depolarizing drive for action potential regeneration and were hence important in regulating neuronal excitability (158). DRG neurons demonstrated increased excitability in inflammatory conditions. Following reports of inflammatory mediators like bradykinin-influencing Na_V_1.7-generated resurgent currents, Tan *et al*. (158) investigated the role of PKC in this phenomenon, since it participated in signal transduction cascades initiated by several inflammatory agents. Mutation of S1479 in the domain III-IV linker near the inactivation gate to phosphomimetic residues aspartate/glutamate imparts greater resurgent activity to the channel, while the corresponding alanine mutant retained the properties of the WT channel.

### Na_V_1.8

Both decreases and increases in Na_V_1.8 current have been reported in response to PKC (147). The novel isoform, PKCε, has been found to enhance tetrodotoxin (TTX)-insensitive Na_V_1.8 currents in DRG. This effect was initiated *via* neurokinin-1 (NK-1) receptor signaling, potentially exacerbated in states of inflammation (159).

### Na_V_1.9

Gain of function variants of this channel were implicated in familial pain disorders. PKC activation was shown to dose-dependently enhance TTX-resistant (TTX-R) currents (160). Additionally, TTX-R currents in DRG neurons were diminished after treatment with the PKC inhibitor, staurosporine (160). Hyperalgesia in afferent nociceptors *in vivo* was observed through the enhancement of TTX-R currents through Na_v_1.9 by inflammatory agents. TTX-R current through the Na_v_1.9 channel was enhanced by epinephrine. This increase could be reversed by the application of the PKC blocker BIM. εV1–2, a PKCε inhibitory peptide, diminished mechanical hyperalgesia in animals tested in epinephrine-induced mechanical hyperalgesia and carrageenan-induced inflammatory hyperalgesia models. The PKCε inhibitory peptide also halved the epinephrine-induced increase in TTX-R currents, providing evidence of a direct effect of the nPKC, PKCε, on Na_v_1.9 channels (161).

## Epithelial Na^+^ channel

Epithelial Na^+^ channel (ENaC) is a sodium channel that plays a crucial role in sodium homeostasis and in turn total body water regulation. This channel is composed of a combination of three homologous subunits, α, β, and γ (162). The β and γ subunits, but not α, were phosphorylated by PKC in heterologous systems (163). These data are confirmed by Stockand *et al*. (162) who further observed a decrease in membrane expression of the β and γ subunits in heterologous systems due to PKC phosphorylation while the α subunit remained insensitive to PKC. Phosphoinositide regulation of ENaC channels has been studied extensively and shown to be a critical regulatory mechanism of channel activity (164). Direct interactions of PI(4,5)P_2_ with the β and γ subunits of the heterotrimeric ENac channels have been mapped (165), suggesting that PKC effects may be mediated by allosterically modulating channel–PIP_2_ interactions of the β and γ subunits of the channel. This hypothesis remains to be tested.

## Acid-sensing ion channels

Acid-sensing ion channels (ASICs) are sensitive to changes in the proton concentration of the cell. These channels are particularly important in nociception and contribute to mechanosensitivity as well as acid sensing. These channels primarily pass sodium ions with some permeation of calcium ions. ASIC channel activation produces a rapidly desensitizing inward current that depolarizes neurons (166).

ASIC subtypes are ASIC 1, 1a, 1b, 2, 2a, 2b, 3, and 4. Three ASIC subunits are required to form a functional channel. Part of the proton-sensing ability of these channels is attributed to pairs of acidic amino acids located at the interface between two subunits. The expression of ASIC1a in heterologous systems was reportedly enhanced by PKC through an NF-κB-mediated pathway (167). PMA and PdBu-mediated activation of cPKCs and nPKCs resulted in an inhibition of acid-evoked channel activity. PKC did not appear to have any effect on membrane expression of ASIC1b. The inhibition of PKC, however, appeared to decrease the amplitude of acid-evoked currents, suggesting the presence of some basal level of phosphorylation. The alanine mutant ASIC1b(S499A) appeared to rescue this effect and returned acid-evoked currents to the WT level. These reports suggest that a dual phosphorylation by PKC play a role in ASIC1b regulation (168). PKC phosphorylates T39 on the N-terminal region of ASIC2a (169). In the hands of the Suh laboratory, who are PIP_2_ experts, no phosphoinositide (PI4P, PI(4,5)P_2_, PI(3,4,5)P_3_) sensitivity of ASIC channels could be demonstrated, even though arachidonic acid stimulated these currents (170). These channels belong to the rare exceptions of ion channels, along with Na_V_ channels, whose dependence on phosphoinositide regulation has not been described in the literature. If this conclusion withstands the test of time, the PKC-mediated effects seen with ASIC channels may employ mechanisms independent of allosteric modulation of channel–PIP_2_ interactions.

## Conclusions

Ion channel proteins are targets of PKC phosphorylation, a regulatory step with great implications in health and disease. Yet, many studies do not consider that there are 10 PKC isoforms, which phosphorylate distinct sites and that the phosphorylation events are transduced *via* distinct pathways to regulate channel activity. Effects described in the literature often involve channel gating, while in other cases they involve changes in channel expression or trafficking.

PIP_2_ has emerged as a master regulator of ion channel gating. A few examples of PKC-mediated phosphorylation exerting its effects through allosteric modulation of channel–PIP_2_ interactions have been described. In this review, we examined the evidence of PKC modulation of multiple ion channels and their phosphoinositide dependence in parallel. A recent example of utilizing sensitive optogenetic tools to assess whether a known PKC-phosphorylated site exerts its effects *via* PIP_2_ is illustrated with the inhibition of the TRPC5(T972) channel site. A similar approach can be taken with the many sites enumerated in this review. Additionally, novel sites for regulation by specific PKC isoforms need to be identified and tested for allosteric modulation of channel–PIP_2_ interactions. In the cases where atomic resolution structures of PKC-regulated ion channels are known, knowledge of a specific phosphorylation site and its allosteric coupling to the PIP_2_-gating machinery can proceed to MD simulations and elucidation of the dynamic mechanism of gating. This unprecedented potential of mechanistic clarity promises to inform small molecule drug discovery efforts to promote health and suppress disease in cases where PKC regulation is involved.

## Conflict of interest

The authors declare that they have no conflicts of interest with the contents of this article.
